# Vascular Resection, Reconstruction, and Divestment in Pancreatoduodenectomy: Expanding Boundaries in Pancreatic Cancer Surgery

**DOI:** 10.3390/cancers18040577

**Published:** 2026-02-10

**Authors:** Dimitrios Moris, Brian M. Nguyen, Alexander Kroemer, Benjamin Weinberg, Keith R. Unger, Nadim G. Haddad, Thomas M. Fishbein, Yuri S. Genyk

**Affiliations:** 1 Department of Surgery, MedStar Georgetown Transplant Institute, Medstar Georgetown University Hospital, Washington, DC 20007, USA; brian.m.nguyen@gunet.georgetown.edu (B.M.N.); yuri.s.genyk@medstar.net (Y.S.G.); 2 Lombardi Comprehensive Cancer Center, Washington, DC 20007, USA; 3 Radiation Oncology, MedStar Georgetown University Hospital, Washington, DC 20007, USA; 4 Department of Gastroenterology, Medstar Georgetown University Hospital, Washington, DC 20007, USA

**Keywords:** pancreatoduodenectomy, Whipple procedure, vascular resection, portal vein, superior mesenteric vein, arterial resection, arterial divestment, locally advanced pancreatic cancer

## Abstract

Vascular resection and reconstruction during pancreatoduodenectomy (PD) have evolved from rare and controversial procedures into essential components of surgical management for selected patients with locally advanced pancreatic ductal adenocarcinoma (PDAC). Venous resection is now widely accepted and routinely performed in high-volume centers, whereas arterial resection and artery-sparing divestment remain selectively applied because of their technical demands and concerns regarding perioperative risk and oncologic benefit. Accumulating contemporary evidence indicates that venous resection can be performed with acceptable safety, with 30-day mortality rates generally ranging from 3% to 5% and median overall survival of approximately 18–26 months when margin-negative (R0) resection is achieved. Arterial resections, most commonly involving the common hepatic, celiac, or superior mesenteric arteries, have been increasingly utilized in highly selected patients, particularly following neoadjuvant therapy, achieving R0 resection rates of approximately 65–75% and median overall survival of 20–28 months. Arterial divestment has emerged as a promising artery-sparing strategy, offering comparable oncologic outcomes with reduced surgical morbidity in appropriately selected cases. Collectively, these advances have expanded the boundaries of resectability in PDAC, enabling surgical intervention in patients previously deemed inoperable. Venous resection is now considered an oncologically sound extension of standard PD, whereas arterial resection and divestment should remain restricted to carefully selected patients demonstrating favorable biologic behavior and response to neoadjuvant therapy. Future progress in this field will likely depend on improved biologic stratification, enhanced intraoperative perfusion assessment, and the integration of hybrid open and endovascular techniques.

## Simple Summary

Pancreatic ductal adenocarcinoma is an aggressive cancer that often involves major blood vessels, limiting surgical options. Advances in surgical techniques now allow safe vascular resection and reconstruction in selected patients undergoing pancreaticoduodenectomy. Venous resection has become standardized in experienced centers, offering acceptable complication rates and meaningful survival when complete tumor removal is achieved. Arterial resection remains technically demanding and is associated with higher risks, but may benefit carefully selected patients who respond to neoadjuvant therapy. Emerging strategies such as arterial divestment aim to preserve arterial integrity while maintaining oncologic effectiveness. This review summarizes the evolution of vascular surgical approaches in pancreatic cancer, highlights clinical indications for venous and arterial interventions, and provides practical recommendations to guide multidisciplinary decision-making. Continued research is needed to refine patient selection, optimize perioperative management, and improve long-term outcomes.

## 1. Introduction

Pancreatic ductal adenocarcinoma (PDAC) remains one of the deadliest malignancies worldwide, accounting for over 495,000 deaths annually. Only 15–20% of patients present with resectable disease at diagnosis [1]. The overall 5-year survival remains below 12%, largely due to late presentation, early systemic dissemination, intrinsic chemoresistance, and a profoundly immunosuppressive tumor microenvironment [2]. PDAC is characterized by aggressive local invasion, dense desmoplastic stroma, perineural spread, and early vascular encasement, which together contribute to both surgical complexity and poor oncologic outcomes [2]. Vascular encasement of the portal–superior mesenteric vein (PV/SMV) or arterial structures historically precluded curative resection. However, the combination of modern neoadjuvant therapy—most notably FOLFIRINOX—and advances in vascular surgery have redefined the concept of resectability.

Early series from the 1970s and 1980s described portal vein resection (PVR) as technically feasible but oncologically futile. By the 2000s, work from Tseng et al. [2] and Nakao et al. [3] demonstrated that margin-negative PD with venous resection (PDVR) could yield survival equivalent to that of standard PD. Since then, vascular resection has evolved from a sign of desperation to a marker of surgical sophistication.

Despite these advances, arterial involvement—particularly of the common hepatic artery (CHA), celiac axis (CA), or superior mesenteric artery (SMA)—remains contentious. Arterial resections entail longer operative times, increased ischemic complications, and historically poor survival [4,5]. Yet, as systemic therapy has improved, biologically favorable patients have emerged who benefit from such extended resections [6]. More recently, “arterial divestment,” or sub-adventitial dissection of the tumor from arterial surfaces, offers an intermediate solution with reduced morbidity [7].

As multimodal therapy increasingly downstages locally advanced tumors, extended resections incorporating venous and, in highly selected cases, arterial reconstruction have emerged as viable strategies to achieve margin-negative (R0) resection in biologically favorable disease. These developments provide the foundation for the present narrative review, which examines the evolving role of vascular resection, reconstruction, and arterial divestment in pancreatoduodenectomy.

## 2. Venous Resection and Reconstruction

The concept of en bloc resection of the pancreatic head with the portal vein was introduced by Child in 1952, but it was not until Fortner’s “regional pancreatectomy” in the 1980s that vascular reconstruction gained traction [8]. Outcomes remained poor until improvements were made in perioperative management and selective patient inclusion in the modern era. Venous involvement usually occurs at the PV/SMV confluence, extending variably toward the splenic vein or first jejunal branch. The International Study Group of Pancreatic Surgery (ISGPS) classifies resections into four types [9]:

Type I, tangential resection with venorrhaphy;

Type II, segmental resection with primary end-to-end anastomosis;

Type III, segmental resection with interposition graft;

Type IV, complex resection involving multiple tributaries.

**Figure 1:**
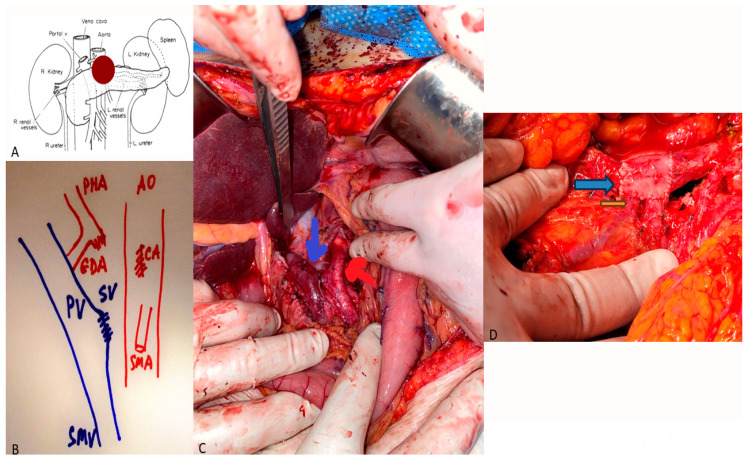
(**A**) Locally advanced pancreatic cancer involving the side wall of the portal vein (PV) and the celiac artery (CA). (**B**) Illustration of the anatomy after resection of the pancreatic mass. Note that the CA is sacrificed and the arterial flow to the liver is maintained via retrograde flow from the superior mesenteric artery (SMA) via an intact gastroduodenal artery (GDA). The PV has been oversewn at the level of the splenic vein (SV). SMV stands for superior mesenteric vein and AO stands for aorta. (**C**) Intraoperative illustration of the anatomy described in (**B**). The blue arrow highlights the venous reconstruction and the red arrow highlights the arterial anatomy. (**D**) Another case of portal vein reconstruction with a left renal vein conduit. The blue arrow highlights the left renal vein conduit and the orange arrow highlights the reimplantation of the inferior mesenteric vein and the splenic vein to the left renal vein conduit.

Primary end-to-end anastomosis is preferred when tension-free approximation is feasible. For longer defects (>3 cm), autologous grafts such as the internal jugular, external iliac, or left renal vein are favored (Figure 1). Prosthetic grafts (PTFE, ringed Gore-Tex) provide comparable patency but may increase infection risk [10]. Modern series consistently demonstrate that venous resection adds minimal risk when performed in high-volume centers. A meta-analysis of 22 studies (*n* = 5488) by Costa et al. [11] found a pooled 30-day mortality of 3.9%, comparable to that of standard PD (3.1%). Major morbidity ranged from 30 to 40%. Venous thrombosis occurred in approximately 7% of cases, predominantly within 30 days, and was more frequent with prosthetic grafts. Hackert et al. [6] reported on 480 patients undergoing PDVR, with 34% major morbidity and 4.1% mortality. Early graft patency exceeded 90% for primary repairs and 82% for graft interpositions. Similarly, Capussotti et al. [12] found no significant difference in morbidity between PDVR and standard PD (*p* = 0.42).

Margin status remains the primary determinant of survival [13]. In the Heidelberg experience, the median OS after PDVR was 25 months for R0 versus 14 months for R1 resections [14]. Tseng et al. [2] observed a 5-year survival of 17% for PDVR, equivalent to that of 18% for standard PD. Across contemporary meta-analyses, the median OS after PDVR ranges from 18 to 26 months, with 5-year survival approaching 20% when R0 resection is achieved [11,15,16]. Neoadjuvant therapy substantially improves outcomes: Beane et al. [17] reported a median OS of 21.3 months after FOLFIRINOX + PDVR compared with 14.2 months for upfront resection. The one-year patency exceeds 85% for autologous grafts and 75% for prosthetic conduits [18]. Re-thrombosis beyond six months rarely impacts liver function owing to collateral development. Radiographic follow-up typically demonstrates compensatory enlargement of the left gastric and coronary veins.

All in all, venous resection and reconstruction during PD are now well-standardized, offering oncologic outcomes comparable to those of conventional resections when performed with negative margins. The incremental operative risk is acceptable, and long-term graft patency is high. The key challenges lie in appropriate selection and ensuring vascular expertise within multidisciplinary pancreatic programs.

## 3. Arterial Resection

Arterial resection in PDAC, historically deemed prohibitive, has re-emerged due to the success of modern neoadjuvant regimens. Early experiences with upfront arterial resections reported mortality rates exceeding 20% [19]. The concept was revived by reports from the Heidelberg, Verona, and Karolinska groups demonstrating feasibility after FOLFIRINOX, with 30-day mortality below 5% [20,21].

**Figure 2:**
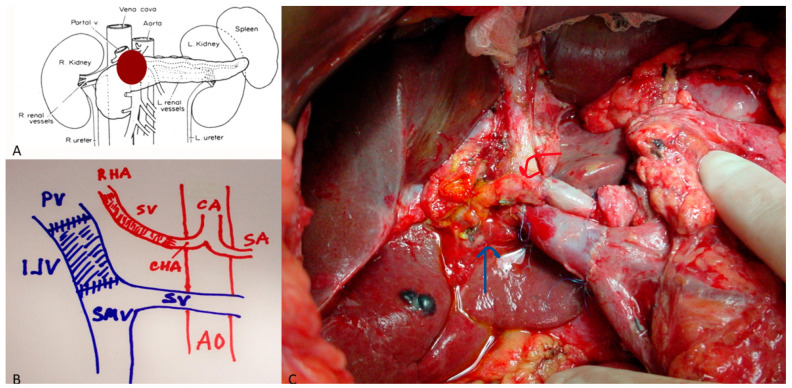
(**A**) Locally advanced pancreatic cancer involving the common hepatic artery (CHA) and the portal vein (PV). (**B**) Illustration of the anatomy after the resection of a pancreatic mass. Note that the CHA has been reconstructed to the right hepatic artery (RHA) with a saphenous vein conduit (SV). The portal vein (PV) has been reconstructed with an internal jugular vein conduit (IJV). The celiac artery (CA), splenic artery (SA), splenic vein (SV) and superior mesenteric vein (SMV) are intact. (**C**) Intraoperative illustration of the anatomy described in (**B**). The blue arrow highlights the venous reconstruction and the red arrow highlights the arterial reconstruction.

**Figure 3:**
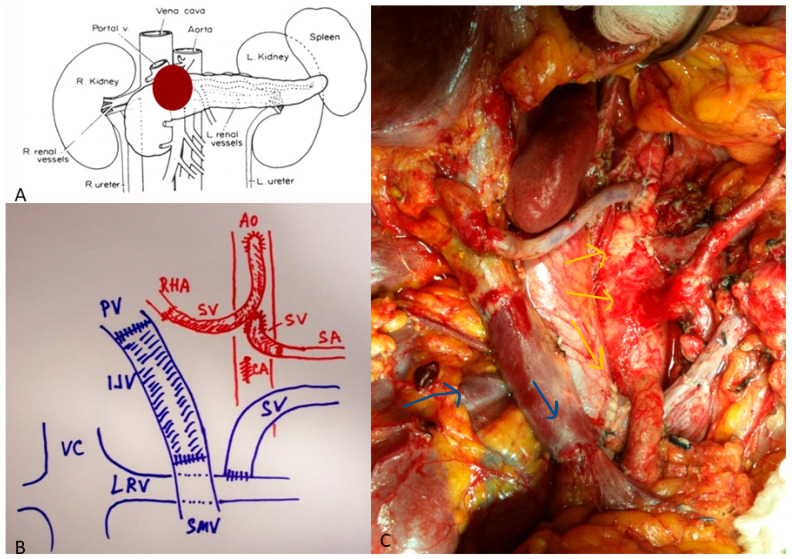
(**A**) Locally advanced pancreatic cancer involving the celiac axis (CA) and the portal vein (PV). (**B**) Illustration of anatomy after the resection of a pancreatic mass. Note that the CA has been totally reconstructed to right hepatic artery (RHA) with a saphenous vein conduit (SV) off the aorta (AO). The splenic artery (SA) has also been reconstructed with the SV conduit. The portal vein (PV) has been reconstructed with an internal jugular vein conduit (IJV). The splenic vein (SV) has been reimplanted to the left renal vein (LRV) with intact outflow to the vena cava (VC). The superior mesenteric vein (SMV) is intact. (**C**) Intraoperative illustration of the anatomy described in (**B**). The blue arrows highlight the venous reconstructions and the yellow arrows highlight the arterial reconstructions.

**Figure 4:**
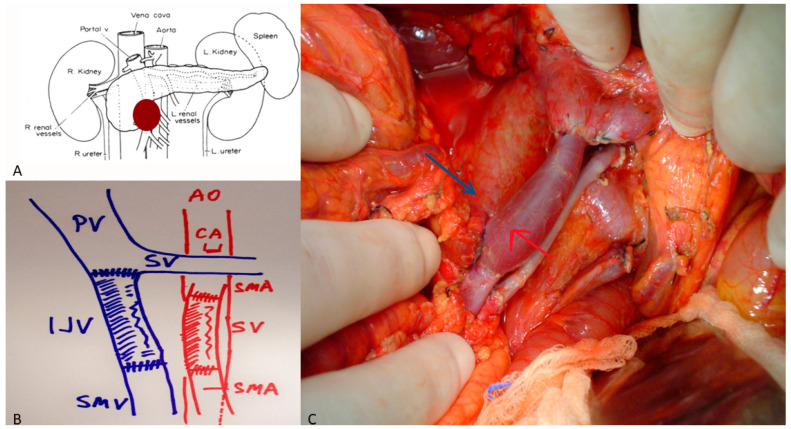
(**A**) Locally advanced pancreatic cancer involving the superior mesenteric artery (SMA) and superior mesenteric vein (SMV). (**B**) Illustration of the anatomy after resection of the pancreatic mass. Note that the celiac artery (CA) is intact off the aorta (AO). The splenic vein (SV) is intact merging to the portal vein (PV), which is also intact. The SMV has been reconstructed with an internal jugular vein conduit (IJV). The SMA has been replaced with a saphenous vein (SV) conduit. (**C**) Intraoperative illustration of the anatomy described in (**B**). The blue arrow highlights the venous reconstruction and the red arrow highlights the arterial reconstruction.

Arterial resections involve the CHA, CA, or SMA. Resection of the CHA can often be repaired primarily or reconstructed using transposition from the splenic or left gastric artery (Figure 2). CA resections, performed mainly during modified Appleby procedures, may require arterial re-routing via the pancreatoduodenal arcade or splenic transposition (Figure 3). SMA reconstruction demands microsurgical precision and is reserved for short segment involvement (Figure 4). Autologous saphenous or radial artery grafts are preferred; prosthetic grafts are used only in exceptional cases [22].

Across 18 modern series encompassing more than 600 arterial resections, the pooled 30-day mortality was 4.7% and the major morbidity was 46%. Post-operative hemorrhage occurred in 8–10% and ischemic complications in 6% [23,24]. The mean operative time was 560 ± 120 min, with a mean blood loss of 1.8 L. Kluger et al. [21] reported a 30-day mortality of 4.5% and a median OS of 22 months in a multi-institutional cohort. R0 resection remains critical: pooled analysis from 10 studies revealed a median OS of 26.4 months for R0 versus 14.1 months for R1 resections (*p* < 0.01). Local recurrence rates are 20–25%, similar to those of venous reconstructions. Nodal positivity is frequent (≈70%), but not independently predictive once R0 margins and neoadjuvant therapy are accounted for [25].

## 4. Arterial Divestment

Arterial divestment—or artery-sparing dissection—entails meticulous sub-adventitial separation of the tumor from major arteries after robust response to neoadjuvant therapy. This avoids vascular anastomosis while achieving circumferential clearance. First described by Nakao and later standardized by Del Chiaro et al. [25], divestment occupies a conceptual middle ground between standard resection and full arterial reconstruction. Arterial divestment can achieve R0 in 77% of cases with a 30-day mortality of 3.9% and a median OS of 26 months—comparable to the outcomes of formal arterial resection but with 50% lower vascular complication rates. Ideal candidates exhibit partial arterial contact (<180°) on post-therapy imaging and normalization of CA19-9 [26]. Frozen-section assessment of the adventitial plane is recommended. Intra-operative fluorescence angiography assists in assessing perfusion after extensive adventitial stripping.

Table 1 and Table 2 summarize the pooled results from venous, arterial, and divestment series.

Venous resections demonstrate the lowest morbidity with equivalent survival; arterial resections carry higher morbidity but remain viable after neoadjuvant therapy. Divestment provides a favorable balance of safety and oncologic efficacy.

## 5. Role of Radiation Therapy in Patients Undergoing Vascular Resection or Reconstruction

The role of radiation therapy (RT)—whether preoperative, postoperative, or intraoperative—has evolved significantly in the multidisciplinary management of PDAC with vascular involvement. While surgery remains the cornerstone of curative intent, RT can improve local control and margin negativity, especially in cases requiring vascular resection or reconstruction.

### 5.1. Preoperative Chemoradiation and SBRT

Preoperative chemoradiation has been associated with higher rates of R0 resection and improved locoregional control in resectable and borderline-resectable PDAC. The landmark PREOPANC randomized trial demonstrated that neoadjuvant gemcitabine-based chemoradiotherapy (36 Gy) resulted in a significantly higher R0 rate (71% vs. 40%) and improved long-term survival compared with upfront surgery, particularly among borderline cases [27,28].

Similarly, systematic reviews and meta-analyses of patients treated with multiagent induction chemotherapy (most commonly FOLFIRINOX) followed by consolidative RT showed increased rates of pathologic response and R0 resection, though the overall survival (OS) advantage remains inconsistent [29,30].

Stereotactic body radiotherapy (SBRT) provides highly conformal, dose-escalated treatment to the tumor–vessel interface with short treatment courses. Multi-institutional analyses have shown that SBRT following chemotherapy achieves R0 rates exceeding 80% and a median OS approaching 25–30 months in selected responders [31,32]. SBRT may thus sterilize the vascular margin (“vascular clinical target volume” or vascular-CTV), converting anatomically borderline or even locally advanced tumors into candidates for curative resection [33,34].

### 5.2. Impact of Radiation on Vascular Reconstruction

Radiation can improve oncologic clearance at the vascular margin but introduces technical complexity. Several studies have reported increased perivascular fibrosis and adhesions, which may complicate venous or arterial reconstruction. Portal vein stenosis following preoperative chemoradiation has been observed in up to 30–40% of highly selected cohorts, though most cases remain asymptomatic [35]. Preoperative planning should therefore include careful vascular imaging and multidisciplinary review to balance oncologic benefit and operative risk.

Recent dosimetric analyses emphasize that adequate vascular-CTV dosing is critical. Delivering an equivalent dose (EQD2 ≥ 30–33 Gy) to the tumor–vessel interface has been correlated with improved local control and reduced vascular recurrence [36]. These findings highlight the need for deliberate inclusion of perivascular structures in RT planning for patients expected to undergo vascular resection.

### 5.3. Intraoperative Radiotherapy (IORT)

IORT enables the delivery of a focused, high-dose boost directly to the tumor bed or vascular margin during resection. Systematic reviews suggest that IORT improves local control and, in some series, overall survival without increasing postoperative morbidity [37,38]. Contemporary experiences with low-energy X-ray IORT devices confirm its technical feasibility and safety in selected patients [39]. ESTRO/ACROP consensus guidelines now endorse IORT as an adjunct in cases where the posterior or vascular margins remain at risk [40].

Taken together, radiation—whether external beam, SBRT, or IORT—has a meaningful role in optimizing outcomes for patients undergoing vascular resection or reconstruction for PDAC. It can increase the probability of margin-negative resection and reduce local failure, albeit at the cost of greater technical difficulty during surgery. Integration of RT should therefore be individualized within a multidisciplinary setting, balancing oncologic benefit and reconstructive feasibility, as summarized in Table 3.

## 6. Discussion

Vascular resection has transformed PD from a purely resective procedure into a multidisciplinary onco-vascular operation. The principal lesson from two decades of experience is that biology trumps anatomy. While early efforts focused on technical feasibility, current practice integrates biological selection through neoadjuvant therapy response, radiologic regression, and serum biomarker normalization.

Venous resection no longer confers a survival penalty [5,6,11,13,25]. The major survival driver is R0 resection after systemic therapy. The convergence of survival between venous and standard PD reflects the normalization of case selection and surgical expertise. In contrast, arterial resection and divestment serve as salvage strategies for responders who would otherwise be unresectable; their 2-year survival is approximated at 45%—comparable to that of metastatic patients achieving complete response on FOLFIRINOX [7,20,23,24,25,26]. Most available data derive from retrospective, single-institution experiences, subject to selection bias and heterogeneous definitions of vascular involvement. Few randomized trials exist; the Japanese JASPAC and French PRODIGE 48 studies are ongoing. The absence of standardized reporting for divestment further complicates comparisons.

The future of vascular PD lies in individualized therapy—matching biological response with the minimal necessary vascular intervention. Multicenter registries and prospective trials with standardized reporting of vascular involvement and reconstruction type are needed. The distinction between arterial resection and divestment will likely blur as precision imaging delineates true adventitial invasion. The evolving role of vascular resection and reconstruction in pancreatic surgery reflects not only technical progress but also a deeper philosophical shift in how we define resectability. Historically, vascular involvement signified the limits of surgical intervention; today, it increasingly marks the limits of our multidisciplinary coordination and systemic therapy. The question is no longer if we can perform vascular resection, but when and for whom should it be performed.

To translate available evidence into clinical practice, several pragmatic recommendations can be proposed. Venous resection involving the PV/SMV should be considered a standard extension of pancreatoduodenectomy when R0 resection appears achievable and the procedure is performed in high-volume centers with vascular expertise. Venous involvement alone should not preclude surgical exploration in patients demonstrating stable or responsive disease after neoadjuvant therapy. Arterial resection should be reserved for highly selected patients, ideally those who have completed neoadjuvant therapy with radiographic disease stability or regression, normalization or a marked decline in CA19-9, and the absence of metastatic progression. These procedures should be limited to centers with advanced hepatopancreatobiliary and vascular surgical capability, given the elevated risk of ischemic and hemorrhagic complications. Arterial divestment (artery-sparing peeling) may be preferable in cases of partial arterial contact (<180°) where imaging and intraoperative assessment suggest the absence of full-thickness arterial wall invasion. This strategy offers a balance between oncologic radicality and surgical safety, potentially reducing morbidity while preserving survival benefit. Finally, multidisciplinary tumor board evaluation is essential in determining candidacy for vascular intervention, integrating surgical, medical oncology, radiation oncology, radiology, and vascular perspectives. Emerging tools such as intraoperative fluorescence angiography and perfusion assessment may further refine intraoperative decision-making regarding arterial sacrifice or preservation (Table 4).

Despite growing experience, major knowledge gaps persist regarding the optimal extent of vascular intervention in PDAC. Most available data derive from retrospective, single-institution series subject to selection bias, heterogeneous neoadjuvant protocols, and inconsistent definitions of vascular involvement. Prospective evidence guiding patient selection, procedural choice, and oncologic sequencing remains limited. Future research should prioritize prospective multicenter cohorts and registry-based studies to standardize reporting of venous resection, arterial reconstruction, and arterial divestment. Comparative effectiveness studies are needed to determine whether arterial divestment offers a superior risk–benefit balance relative to formal arterial resection in borderline and locally advanced tumors. Additional priorities include the development of molecular, radiomic, and biomarker-based predictors to identify patients most likely to benefit from aggressive vascular surgery. Integration of translational endpoints into surgical trials may enable biologic stratification beyond anatomic criteria alone. Prospective trials should also evaluate novel intraoperative perfusion-monitoring technologies, hybrid open–endovascular approaches, and the role of targeted radiation to the tumor–vessel interface. International collaborative consortia and standardized data-sharing frameworks will be essential to transition vascular reconstruction in PDAC from expert-driven practice to evidence-based oncologic strategy.

## 7. Conclusions

In conclusion, venous resection should be regarded as a standardized component of modern pancreatoduodenectomy, while arterial resection and divestment should be selectively applied based on biological response, technical feasibility, and institutional expertise. Future progress will depend on rigorous prospective research, improved biologic selection, and coordinated multidisciplinary innovation.

## Figures and Tables

**Table 1:** Comparative outcomes of vascular techniques in pancreatoduodenectomy.

Technique	No. of Studies	Patients (*n*)	30-Day Mortality (%)	Major Morbidity (%)	R0 (%)	Median OS (mo)
Venous resection (PV/SMV)	25	>5000	3.5	36	78	22
Arterial resection (CHA/CA/SMA)	18	>600	4.7	46	73	24
Arterial divestment	7	>400	3.8	29	77	26

**Table 2:** Techniques and materials for vascular reconstruction.

Vessel	Preferred Technique	Graft/Material	1-yr Patency (%)	Key References
PV/SMV	End-to-end anastomosis	—	>90	[12,13]
PV/SMV (long defect)	Interposition graft	Autologous (IJV, EIV, left renal vein) > PTFE	85 vs. 75	[11,16]
CHA	Primary repair or transposition	Splenic or Left gastric artery	—	[21,23]
CA	Modified Appleby with re-routing	Pancreatoduodenal arcade flow	—	[18,19]
SMA	Autologous artery graft	Saphenous vein or Radial artery	80	[21,25]

**Table 3:** Role of radiation therapy in patients with pancreatic cancer undergoing or considered for vascular resection/reconstruction.

Study (Year)	Design/Population	Treatment Regimen	Main Findings
PREOPANC Trial [27]	Randomized phase III, 246 resectable/borderline PDAC	Neoadjuvant gemcitabine-based chemoradiotherapy (36 Gy) vs. upfront surgery	Higher R0 rate (71% vs. 40%); improved 5-yr OS (20% vs. 6%); strongest effect in borderline disease
Suker et al., 2016 [29]	Systematic review and meta-analysis	Induction FOLFIRINOX ± RT	R0 resection 28%; OS 24 mo in resected cases
Janssen et al., 2021 [30]	Meta-analysis	Neoadjuvant FOLFIRINOX ± RT	RT increased pathologic response and R0 rate; OS benefit unclear
Hill et al., 2022 [31]	Multicenter retrospective, borderline/locally advanced PDAC	SBRT after chemotherapy	R0 > 85%; median OS 27 mo; low toxicity
Mellon et al., 2016 [33]	Single-center series, 88 patients	Induction chemo + SBRT vs. upfront surgery	Comparable or improved perioperative outcomes; OS ≈ 30 mo
Nelson et al., 2022 [36]	Multi-institutional, 143 patients	Neoadjuvant chemoradiation with vascular CTV delineation	Higher dose to vascular CTV → better local control
Jin et al., 2020 [37]	Systematic review and meta-analysis	Intraoperative radiotherapy (IORT)	Improved local control and OS; no increase in major morbidity
Krempien and Roeder, 2017 [38]	Narrative review/expert consensus	IORT ± EBRT	Safe and feasible; recommended for posterior or vascular margins
Cho et al., 2022 [39]	Prospective series, 29 patients	Low-energy X-ray IORT during resection	No increase in complications; promising local control

Abbreviations: PDAC = pancreatic ductal adenocarcinoma; SBRT = stereotactic body radiotherapy; IORT = intraoperative radiotherapy; CTV = clinical target volume; OS = overall survival; R0 = margin-negative resection.

**Table 4:** Practical clinical recommendations for venous resection, arterial resection, and arterial divestment during pancreatoduodenectomy.

Clinical Scenario	Recommended Strategy	Key Selection Criteria	Rationale	Setting/Expertise Required
PV/SMV abutment or short-segment involvement with reconstructable vein	Venous resection and reconstruction (standard PD extension)	Potential for R0 resection; no distant metastasis; acceptable performance status	Comparable survival to standard PD; low incremental morbidity in experienced centers	High-volume pancreatic center with vascular expertise
Long-segment venous involvement requiring graft	Venous resection with autologous interposition graft	Good response or stable disease after neoadjuvant therapy; preserved liver inflow/outflow	Enables oncologic clearance while maintaining venous patency	HPB + vascular surgery program
Arterial abutment or encasement after neoadjuvant therapy with biologic response	Selective arterial resection and reconstruction	Radiographic stability/regression; CA19-9 decline; no progression; good physiologic reserve	Potential for R0 resection in biologically favorable disease	Ultra–high-volume center with arterial reconstruction capability
Partial arterial contact (<180°) without full-thickness invasion	Arterial divestment (artery-sparing peeling)	Favorable imaging; intraoperative confirmation; frozen-section negativity	Preserves arterial integrity; lowers ischemic and bleeding risk	Expert pancreatic surgeon with vascular experience
Poor response to neoadjuvant therapy or radiographic progression	Avoid arterial resection; consider systemic therapy or palliation	Rising CA19-9; disease progression; poor performance status	High risk of futile surgery and early recurrence	Multidisciplinary tumor board decision
Borderline vascular involvement with uncertain perfusion	Use intraoperative perfusion assessment (e.g., ICG angiography)	Indeterminate arterial flow or collateralization	Reduces ischemic complications; informs reconstruction decisions	Advanced intraoperative imaging availability

## Data Availability

No new data were created.
